# Advances of mRNA vaccines for COVID-19: A new prophylactic revolution begins

**DOI:** 10.1016/j.ajps.2021.02.005

**Published:** 2021-03-22

**Authors:** Yuhua Weng, Yuanyu Huang

**Affiliations:** Institute of Engineering Medicine, Advanced Research Institute of Multidisciplinary Science, School of Life Science, Key Laboratory of Molecular Medicine and Biotherapy, Beijing Institute of Technology, Beijing 100081, China

## Abstract

**Figure:**
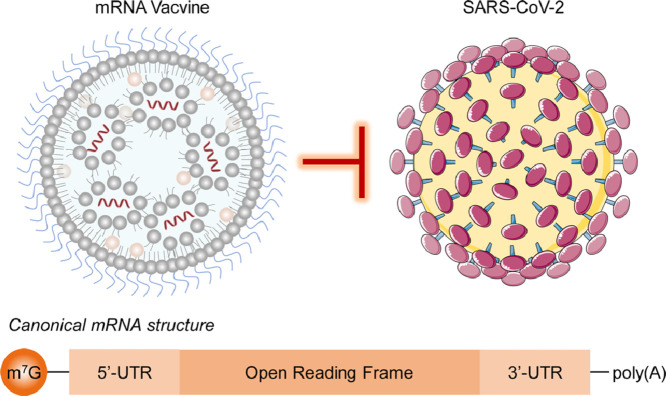

The world is in the midst of 2019 coronavirus infection disease (COVID-19) pandemic. As of 30 January 2021, more than 100 million cases and 2.1 million deaths were confirmed according to the data from the World Health Organization (WHO), resulting in a widespread social and economic turmoil. Therefore, researches worldwide are racing to deploy safe and effective COVID-19 vaccines.

Multiple approaches have been used to develop COVID-19 vaccines, including inactivated, attenuated virus vaccines, viral proteins, DNA, RNA vaccines and virus-like particles [1]. To date, the WHO has documented more than 290 COVID-19 vaccine candidates, with four of them are now being licensed and authorized for use in some regions, including two mRNA vaccines (BNT162b2 developed by BioNTech/Pfizer [2] and mRNA-1273 developed by Moderna/National Institute of Allergy and Infectious Diseases (NIAID) [3]). They both targeted the same virus antigen, the spike protein (S protein) with two proline substitutions (S-2P) at residues K986 and V987. The S-2P protein retains the S protein of SARS-CoV in its prefusion conformation and induces higher titers of neutralizing antibodies (nAbs) than wild-type S protein in mice [4], [5], [6], [7].

According to the published results from phase 3 clinical trials of the two mRNA vaccines, all the participants received two-dose mRNA injections with a median follow-up time of 2 months after the second dose. For BNT162b2, only 8 cases of COVID-19 after the second dose were observed among vaccine recipients, as compared with 162 among placebo recipients, for an overall efficacy of 94.6%. Between the first dose and the second dose, the BNT162b2 still resulted in a vaccine efficacy of 52%. For the primary analysis of mRNA-1273, 11 COVID-19 cases in the vaccine group and 185 cases in the placebo group were diagnosed, indicating 94.1% efficacy of the vaccine. The vaccine efficacy to prevent COVID-19 was consistent across subgroups of age, presence of risk for severe COVID-19, sex and race. No severe side effects happened in clinical trials, the frequent side effects of the two mRNA vaccines included moderate pain at the injection site, fatigue and headache [2,3,8]. The results were highly impressive. Why were the mRNA vaccines produced from scratch in such a short time and so effective?

The formulation of mRNA vaccine is mainly composed of two parts: mRNA encoding the antigen and lipid nanoparticles encapsulating the mRNA. Natural mRNA has a single strand structure, consisting of 5′-cap, poly (A) tail, protein encoding open reading frame (ORF) and untranslated regions (UTRs) (Fig. S1) [9,10]. In order to improve the druggability of mRNA, a variety of chemical modifications to mRNA structures such as adding cap analogue, poly (A), modified nucleosides, etc. were explored. To date, several mRNA modalities are developed for infectious disease vaccination, including the uridine-containing mRNA (uRNA), nucleoside modified mRNA (modRNA), self-amplifying mRNA (saRNA) and trans-amplifying mRNA (taRNA). The saRNA resembles canonical mRNA encoding the protein of interest, but also encoding replicase that multiplies mRNA in the target cell. The taRNA is an advancement platform of saRNA, it enabled scientist to produce the replicase in advance for use with different vaccines, which makes the development of several therapeutic mRNAs at the same time and permitting production of multimeric antigen complexes in a single vaccine become possible.

Besides digging in optimizing the mRNA sequence, employment of proper delivery system is also crucial for mRNA vaccine development, because carrier materials not only can deliver mRNA to the intended site of action, but also protect mRNA from degradation. Among the available options, lipid nanoparticle (LNP) is the most advanced mRNA delivery systems and is employed by almost all COVID-19 mRNA vaccines, including mRNA-1273, BNT162b2, CVnCoV, ARCT-021 and ARCoV (Table S1). The LNP is mainly composed of ionizable lipid, helper lipid, PEG-lipid and cholesterol [9]. LNP is able to encapsulate RNAs in its cavity and form nano-sized vesicles with well-organized lipid structures. It is worth mentioning that three LNP-formulated RNA therapeutics, Onpattro, mRNA-1273, and BNT162b2, have been approved for use worldwide to date, indicating that LNP exhibits superior advantages over other kinds of delivery carriers.

Now we may understand why the mRNA vaccine “hit the line” in the first place. Firstly, design of mRNA sequence is extremely fast and efficient. As soon as Chinese scientists disclosed the complete RNA sequence of this newly-emerged coronavirus to the world, the design of antigen encoding mRNA immediately started. Secondly, unlike producing traditional virus vaccines, which requires more than one year for amplification of cell lines and clinical-grade subunit proteins, manufacturing mRNA can be achieved in a matter of weeks via an *in vitro* transcription process. If the virus mutates significantly and the vaccine becomes ineffective, a new mRNA vaccine can be quickly redesigned and manufactured according to the new virus sequence. Thirdly, in addition to the advantage of manufacturing speed, the immunogenicity of mRNA is highly controllable, which enables mRNA not only to be used in treatment of a variety of antigen-based diseases but also to be employed in protein replacement therapy [9].

Nevertheless, as a newly emerged technology, several important concerns of mRNA vaccines remains to be addressed. The BNT162b2 mRNA vaccine has an extremely strict requirement of −70 °C storage temperature, which brings additional challenges for vaccine transportation and storage. According to the published results from clinical trials, around 20 thousand participants received BNT162b2 or mRNA-1273 injection, respectively [2, 3]. Will side effects or unexpected safety issues arise when the number of people grows to millions and possibly billions? This is the first time mRNA vaccines have ever been marketed and applied worldwide. If the mRNA vaccines prove successful over time, it will not only have huge implications for prevention of COVID-19 (Table S1), but will also revolutionize the development of gene and protein therapeutics.

## Conflicts of interest

The authors report no conflicts of interest.

## Appendix Supplementary materials

Image, application 1
