# Supplementary material for: Advances of mRNA vaccines for COVID-19: A new prophylactic revolution begins
Source: Asian J Pharm Sci. 2021 Mar 22;16(3):263–4. doi: 10.1016/j.ajps.2021.02.005 (PMC8261073; doi:10.1016/j.ajps.2021.02.005)
Supplement: Supplementary file 1 [file mmc1.docx]

**Supporting Information**

**Advances of mRNA vaccines for COVID-19: a new prophylactic revolution begins**

Yuhua Weng ^a^, Yuanyu Huang ^a,*^

^a^ Institute of Engineering Medicine, Advanced Research Institute of Multidisciplinary Science, School of Life Science, Key Laboratory of Molecular Medicine and Biotherapy, Beijing Institute of Technology, Beijing 100081, China.


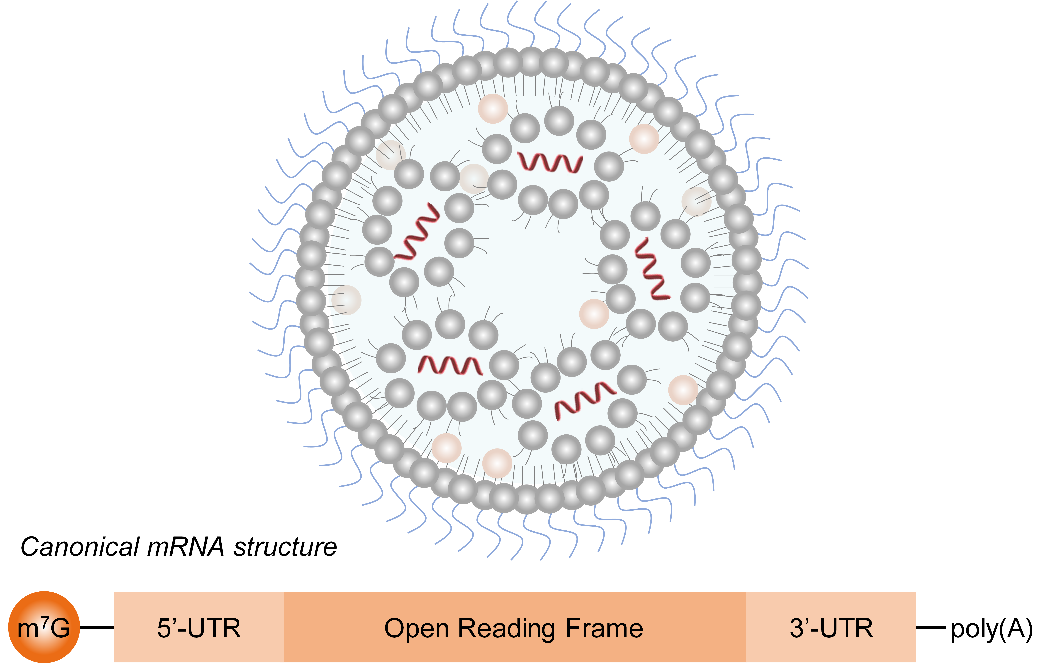


Figure S1 The schematic diagram of LNP-mRNA vaccine

Table S1 mRNA vaccines for COVID-19.

| **Product** | **Delivery** | **Trial ID** | **Phase/Status** | **Start Date** | **Participants** | **Sponsor** | **Location** | **References** |
| --- | --- | --- | --- | --- | --- | --- | --- | --- |
| mRNA-1273 | LNP | NCT04470427 | EUA Approved/Active, not recruiting | 7/27/2020 | ≥18 years old | Moderna Therapeutics | United States | [[1](#_ENREF_1)] |
|  |  | NCT04649151 | III/ Open | 12/9/2020 | 12-18 years old | Moderna Therapeutics | United States |  |
|  |  | NCT04405076 | II/ Closed | 5/25/2020 | 18-54 years old | Moderna Therapeutics | United States |  |
|  |  | NCT04283461 | I/ Closed | 3/16/2020 | 18-99 years old | NIH, NIAID, Moderna Therapeutics | United States | [[2](#_ENREF_2), [3](#_ENREF_3)] |
|  |  | NA (1-year booster study) | I/Planned | 7/1/2021 (Anticipated) | Adult subjects | Moderna Therapeutics | United State |  |
|  |  | NA (Study in young children) | I/Planned | N/A | 1-11 years old | Moderna Therapeutics | United State |  |
| BNT162b2 | LNP | NCT04713553 | EUA Approved/ Not recruiting | 1/28/2021 | 18-55 years old | BioNTech, Pfizer | Germany |  |
|  |  | NCT04588480 | I/II/Not Recruiting | 10/21/2020 | 20-85 years old Japanese | BioNTech, Pfizer | Germany |  |
|  |  | ChiCTR2000040044, NCT04649021 | II/ Open | 11/25/2020 | Chinese healthy subjects | BioNTech, Shanghai Fosun Pharmaceutical (Group) Co. | Germany, China |  |
| BNT162b1, BNT162b2 | LNP | NCT04368728 | II/III/ Active, not recruiting | 4/29/2020 | ≥12 years old | BioNTech, Pfizer | Germany | [[4-6](#_ENREF_4)] |
|  |  | NCT04380701 | I/II/ Open | 4/23/2020 | 18-85 years old | BioNTech | Germany | [[7](#_ENREF_7)] |
| BNT162b3 | LNP | NCT04537949 | I/II/ Open | 9/9/2020 | 18-85 years old | BioNTech | Germany |  |
| BNT162b1 | LNP | ChiCTR2000034825, NCT04523571 | I/ Completed | 7/18/2020 | Healthy Chinese adults | BioNTech, Shanghai Fosun Pharmaceutical (Group) Co. | Germany, China |  |
| CVnCoV | LNP | NCT04674189 | III/ Open | 12/23/2020 | ≥18 years old | CureVac | Germany | [[8](#_ENREF_8)] |
|  |  | NCT04652102, EudraCT Number: 2020-003998-22 | II/III/ Open | 12/14/2020 | ≥18 years old | CureVac | Germany |  |
|  |  | NCT04515147 | II/ Closed | 9/28/2020 | ≥18 years old | CureVac | Germany |  |
|  |  | NCT04449276, EudraCT Number: 2020-001286-36 | I/ Closed | 6/18/2020 | 18-60 years old COVID-19 seropositives | CureVac | Germany | [[9](#_ENREF_9)] |
| ARCT-021 | LNP | NCT04480957 | I/II/ Open | 8/4/2020 | 21-80 years old | Arcturus Therapeutics | United States | [[10](#_ENREF_10), [11](#_ENREF_11)] |
|  |  | NCT04668339 | II/ Open | 1/7/2021 | ≥18 years old | Arcturus Therapeutics | United States |  |
| LNP-nCoVsaRNA | LNP | ISRCTN17072692, EudraCT Number: 2020-001646-20 | I/ Open | 6/16/2020 | 18-75 years old | Imperial College London | United Kingdom | [[12](#_ENREF_12)] |
| ARCoV | LNP | ChiCTR2100041855 | II/ Planned | 1/11/2021 (Anticipated) | Healthy adults | Suzhou Abogen Biosciences Co., Walvax Biotechnology Co. | China, China | [[13](#_ENREF_13)] |
|  |  | ChiCTR2000039212 | I/ Planned | 10/28/2020 (Anticipated) | Healthy subjects | Suzhou Abogen Biosciences Co., Walvax Biotechnology Co. | China, China |  |
|  |  | ChiCTR2000034112 | I/ Planned | 6/25/2020 (Anticipated) | Healthy volunteers | Suzhou Abogen Biosciences Co., Walvax Biotechnology Co. | China, China |  |
| ChulaCov19 | LNP | NCT04566276 | I/ Planned | 1/1/2021 (Anticipated) | Healthy subjects | Mahidol University, Bangkok National Research Council of Thailand | Thailand | [[14](#_ENREF_14)] |
| MRT5500 | LNP | N/A | I/II/ Planned | 3/1/2021 (Anticipated) | N/A | Translate Bio, Sanofi Pasteur | United States, France | [[15](#_ENREF_15), [16](#_ENREF_16)] |
| DS-5670 | LNP | N/A | I/ Planned | 3/1/2021 (Anticipated) | N/A | Daiichi Sankyo | Japan | [[17](#_ENREF_17)] |
| NS | Naked mRNA, with TriMix mRNA adjuvant | N/A | I/ Planned | 3/1/2021 (Anticipated) | Healthy subjects | eTheRNA Immunotherapies, Epivax | Belgium, United States | [[18](#_ENREF_18), [19](#_ENREF_19)] |
| NS (COVID-19 mRNA Vaccine) | Lipid-Polymer Hybrid NP | N/A | I/ Planned | N/A | Healthy subjects (predicted) | Stemirna Therapeutics, Tibet Rhodiola Pharmaceutical Holding Co. | China, China | [[20](#_ENREF_20)] |
| NS | N/A | N/A | I | N/A | N/A | Biocad Biotechnology | Russian Federation | [[21](#_ENREF_21)] |

Abbreviations: LNP, lipid nanoparticle; NP, nanoparticle; i.m., intramuscular injection; i.n., intranasal; N/A, not available; NS, not specified; NIH, National Institutes of Health; NIAID, National Institute of Allergy and Infectious Diseases.

**References**

[1] Baden LR, El Sahly HM, Essink B, et al. Efficacy and Safety of the mRNA-1273 SARS-CoV-2 Vaccine. N Engl J Med 2020; doi:10.1056/NEJMoa2035389.

[2] Jackson LA, Anderson EJ, Rouphael NG, et al. An mRNA Vaccine against SARS-CoV-2 - Preliminary Report. N Engl J Med 2020; 383(20):1920-31.

[3] Anderson EJ, Rouphael NG, Widge AT, et al. Safety and Immunogenicity of SARS-CoV-2 mRNA-1273 Vaccine in Older Adults. N Engl J Med 2020; 383(25):2427-38.

[4] Polack FP, Thomas SJ, Kitchin N, et al. Safety and Efficacy of the BNT162b2 mRNA Covid-19 Vaccine. N Engl J Med 2020; 383(27):2603-15.

[5] Walsh EE, Frenck RW, Jr., Falsey AR, et al. Safety and Immunogenicity of Two RNA-Based Covid-19 Vaccine Candidates. N Engl J Med 2020; 383(25):2439-50.

[6] Mulligan MJ, Lyke KE, Kitchin N, et al. Phase I/II study of COVID-19 RNA vaccine BNT162b1 in adults. Nature 2020; 586(7830):589-93.

[7] Sahin U, Muik A, Derhovanessian E, et al. COVID-19 vaccine BNT162b1 elicits human antibody and TH1 T cell responses. Nature 2020; 586(7830):594-99.

[8] CureVac. Revolutionizing mRNA for Life. https://[www.curevac.com/wp-content/uploads/2021/01/202101_CureVac-JPM-Investor-Handout.pdf](http://www.curevac.com/wp-content/uploads/2021/01/202101_CureVac-JPM-Investor-Handout.pdf) 2021; 1-38.

[9] CureVac. CVnCoV –Positive Interim Phase 1 Data. https://[www.curevac.com/wp-content/uploads/2020/11/20201110-CVAC-WEBCAST-CVnCoV-Positive-Interim-Phase-1-data_final.pdf](http://www.curevac.com/wp-content/uploads/2020/11/20201110-CVAC-WEBCAST-CVnCoV-Positive-Interim-Phase-1-data_final.pdf) 2020; 1-15.

[10] Knezevic I, Liu MA, Peden K, et al. Development of mRNA Vaccines: Scientific and Regulatory Issues. Vaccines (Basel) 2021; 9(2):81.

[11] Arcturus. Arcturus Therapeutics Receives FDA Allowance to Proceed with Phase 2 Study of ARCT-021 (LUNAR-COV19) Vaccine Candidate in the United States. https://ir.arcturusrx.com/node/10351/pdf 2021; 1-2.

[12] Scarabel L, Guardascione M, Dal Bo M, et al. Pharmacological strategies to prevent SARS-CoV-2 infection and to treat the early phases of COVID-19 disease. Int J Infect Dis 2021; 10.1016/j.ijid.2021.01.035.

[13] Zhang NN, Li XF, Deng YQ, et al. A Thermostable mRNA Vaccine against COVID-19. Cell 2020; 182(5):1271-83.e16.

[14] who.int. COVID-19 vaccine. https://vac-lshtm.shinyapps.io/ncov_vaccine_landscape/#. 2021;

[15] Kalnin KV, Plitnik T, Kishko M, et al. Immunogenicity of novel mRNA COVID-19 vaccine MRT5500 in mice and non-human primates. bioRxiv 2020; 10.1101/2020.10.14.337535.2020.10.14.337535.

[16] Bio T. Sanofi and Translate Bio mRNA COVID-19 Vaccine Candidate Induced High Antibody Levels in Preclinical Studies. https://investors.translate.bio/node/7696/pdf 2020; 1-3.

[17] SANKYO D. Global Pharma Innovator with Competitive Advantage in Oncology. https://[www.daiichisankyo.com/files/investors/library/materials/2020/pdf/20210113%E2%80%97JPM%20Conference.pdf](http://www.daiichisankyo.com/files/investors/library/materials/2020/pdf/20210113%E2%80%97JPM%20Conference.pdf) 2021; 1-49.

[18] eTheRNA. eTheRNA Launches an International Consortium and Starts Development of Cross-strain Protective CoV-2 mRNA Vaccine for High Risk Populations. https://[www.prnewswire.com/news-releases/etherna-launches-an-international-consortium-and-starts-development-of-cross-strain-protective-cov-2-mrna-vaccine-for-high-risk-populations-301028630.html](http://www.prnewswire.com/news-releases/etherna-launches-an-international-consortium-and-starts-development-of-cross-strain-protective-cov-2-mrna-vaccine-for-high-risk-populations-301028630.html) 2020; 1-1.

[19] Jong W, Leal L, Buyze J, et al. Therapeutic Vaccine in Chronically HIV-1-Infected Patients: A Randomized, Double-Blind, Placebo-Controlled Phase IIa Trial with HTI-TriMix. Vaccines (Basel) 2019; 7(4):

[20] Stemirna. Stemirna COVID-19 vaccine candidate obtains clinical trial approval in China. https://news.yahoo.com/stemirna-covid-19-vaccine-candidate-015100236.html 2021; 1-1.

[21] BIOCAD. BIOCAD started working on mRNA vaccine against coronavirus. https://biocadglobal.com/index.php?posts&post=45 2020; 1-1.
